# New triazinephosphonate dopants for Nafion proton exchange membranes (PEM)

**DOI:** 10.3762/bjoc.20.145

**Published:** 2024-07-17

**Authors:** Fátima C Teixeira, António P S Teixeira, C M Rangel

**Affiliations:** 1 Laboratório Nacional de Energia e Geologia, I.P., Estrada do Paço do Lumiar, 22, 1649-038 Lisboa, Portugal; 2 Departamento de Ciências Médicas e da Saúde, Escola de Saúde e Desenvolvimento Humano & LAQV- REQUIMTE, IIFA, Universidade de Évora, R. Romão Ramalho, 59, 7000-671 Évora, Portugal

**Keywords:** electrolyser, fuel cells, Nafion-modified membranes, phosphonates, proton exchange membranes, triazine

## Abstract

A new paradigm for energy is underway demanding decarbonized energy systems. Some of them rely on emerging electrochemical devices, crucial in hydrogen technologies, including fuel cells, CO_2_ and water electrolysers, whose applications and performances depend on key components such as their separators/ion-exchange membranes. The most studied and already commercialized Nafion membrane shows great chemical stability, but its water content limits its high proton conduction to a limited range of operating temperatures. Here, we report the synthesis of a new series of triazinephosphonate derivatives and their use as dopants in the preparation of new modified Nafion membranes. The triazinephosphonate derivatives were prepared by substitution of chlorine atoms in cyanuric chloride. Diverse conditions were used to obtain the trisubstituted (4-hydroxyphenyl)triazinephosphonate derivatives and the (4-aminophenyl)triazinephosphonate derivatives, but with these amino counterparts, only the disubstituted compounds were obtained. The new modified Nafion membranes were prepared by casting incorporation of the synthesized 1,3,5-triazinephosphonate (TPs) derivatives. The evaluation of the proton conduction properties of the new membranes and relative humidity (RH) conditions and at 60 °C, showed that they present higher proton conductivities than the prepared Nafion membrane and similar or better proton conductivities than commercial Nafion N115, in the same experimental conditions. The Nafion-doped membrane with compound **TP2** with a 1.0 wt % loading showed the highest proton conductivity with 84 mS·cm^−1^.

## Introduction

Decarbonized energy sources are the new paradigm in a world with increasing energy demands, primarily powered by fossil fuels, being proposed as a key strategy for restricting the detrimental effects of climate change. Vast efforts are being made to fulfil this crucial challenge of the 21st century. Clean, renewable, and environmental-friendly technological processes are being considered using electrochemical devices which convert chemical to electric energy and/or vice versa that, when associated to renewable energy sources, can promote sustainable energy systems [1–4]. Among them are included proton exchange membrane devices [5–6], such as proton exchange membrane fuel cells (PEMFCs) [3,7–9], due to their high-power density and high power-to-weight ratio, and CO_2_ electrolysers, which can reduce the polluting gas CO_2_ and produce syngas from the co-electrolysis of CO_2_ and water [10–11], or water electrolysers that allow the generation of green hydrogen [12–13]. The technology behind these electrochemical devices still relies on the membrane as a key component that defines the applications and the conditions to its use [14]. Both fuel cells and water electrolyser devices depend on proton exchange membranes to ensure a high proton conduction or leading to an efficient reaction production of hydrogen and oxygen gases, with no risk of electrolyte leakage and restricted gas-crossover [15–17].

Also, besides their conductivity and their low permeability to fuel and oxidant, the chemical and structural properties of the membranes restrain their stability and durability, their humidity and temperature application conditions, and their efficiency and consequently, the performance of fuel cells or eletrolysers [18–19].

Many organic polymers with acidic functional groups have been developed as membranes for these electrochemical devices [14,20]. However, many technological limitations remain due to the high dependence of the membrane’s performance on the presence of water or other electrolyte content. To overcome these limitations, the modification of the membrane can be done by the incorporation of other compounds into these polymeric materials to participate in the proton conduction or to surpass the water or electrolyte dependency [15,21].

The most studied and commercially available proton exchange membranes consist of Nafion, a hydrophobic perfluorosulfonated polymer with sulfonic acid groups [22]. These membranes have an excellent chemical stability, but their high proton conduction is dependent of the water content of the membranes which limits their operating temperature range to 80 °C [23].

The importance of the membranes for the new sustainable energy sources fostered the efforts and investments in the research and development of new membranes that might be able to surpass the actual limitations. Our studies started with the synthesis of phosphonate and phosphonic acid compounds to be used as membrane dopants [24–25]. These studies were followed by the incorporation of bisphosphonic acids as dopants in Nafion membranes that led to an increase on the proton conduction of the new membranes, since these compounds are good proton carriers due to their proton donor and acceptor behavior [26–29]. In addition, the increase in the proton conduction of the doped membranes has been shown to be dependent of the chemical structure of the dopant.

Triazines are a class of heteroaromatic compounds with three nitrogen atoms on a six-membered ring, with the general formula C_3_N_3_H_3_. According to the position of the nitrogen atoms in the ring, they constitute 1,2,3-, 1,2,4- and 1,3,5-triazine isomers [30]. There have been reported several and diverse applications to a large number of compounds with a triazine moiety, ranging from biological applications [31–34], such as fungicide, herbicide, antiviral, antimicrobial, antitumor, to their use in organic synthesis, including combinatorial chemistry [35], in analytical chemistry, in electrochemical redox processes, in crystal engineering, and as fluorescent, light emitting, corrosion inhibitors or several other materials [36–42].

The most used triazine is 1,3,5-triazine (or *s*-triazine) that can provide symmetrical monosubstituted derivatives, or di- or trisubstituted symmetrical or asymmetrical derivatives. The *C*_3_ symmetry of 1,3,5-triazine makes it a popular heterocyclic core for the synthesis of star-shaped molecules [43–45], which was being used to the construction of triangular molecules, including molecular cages [46–47] and at porous materials, as linkers in metal-organic frameworks (MOFs) [48], used in the evaluation of the cycloaddition of CO_2_ to epoxides [49] and CO_2_ uptake [50].

Taking in consideration the strategy of the incorporation of dopants to promote the proton conduction in Nafion membranes, new triazinephosphonate (TPs) derivatives were prepared to be applied as dopants through their incorporation into new doped membranes. To this purpose, the present work reports on the synthesis and characterization of a new series of 1,3,5-triazinephosphonate (TPs) derivatives in the anticipation that these dopants can act both as a source of protons and proton acceptors, facilitating the intermolecular proton conduction. The rational of the strategy behind the use of amino- and hydroxyphenyl spacers is twofold: i) their inclusion in the structure separates the bulky phosphonate groups from the triazine moiety, giving the membrane more structural flexibility; ii) nitrogen and oxygen atoms of these groups can also participate in the proton conduction of the membranes. The new membranes were prepared by casting incorporation of the synthesized 1,3,5-triazinephosphonate (TPs) derivatives. Proton conduction properties of new membranes were evaluated by electrochemical impedance spectroscopy (EIS) at 60 °C at different relative humidity (RH) conditions. The results showed higher proton conductivities than recast Nafion membrane and similar or better proton conductivities than commercial Nafion N115, in the same experimental conditions.

## Results and Discussion

### Preparation of triazine derivatives

**Figure 1:**
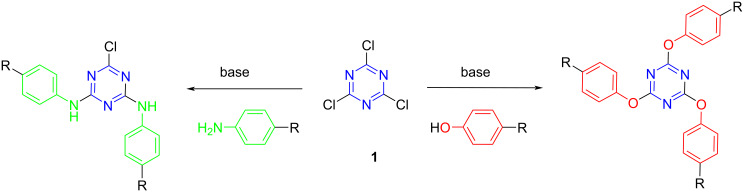
General synthesis of triazinephosphonate compounds.

The synthesis of 1,3,5-triazinephosphonate (TP) derivatives, to be used as dopants on new membranes, were carried out from the commercially available cyanuric chloride (**1**), through the substitution of chlorine atoms by different nucleophiles. The devised strategy involved the attack of the O or N atoms of the arylphosphonate nucleophile at the position of the chlorine atom of triazine, at its 2, 4 and 6 carbon positions (Figure 1). Most of these nucleophiles bearing a phosphonate group were not commercially available and were prepared from 4-hydroxyphenyl- or 4-aminophenyl-based derivatives.

**Scheme 1:**
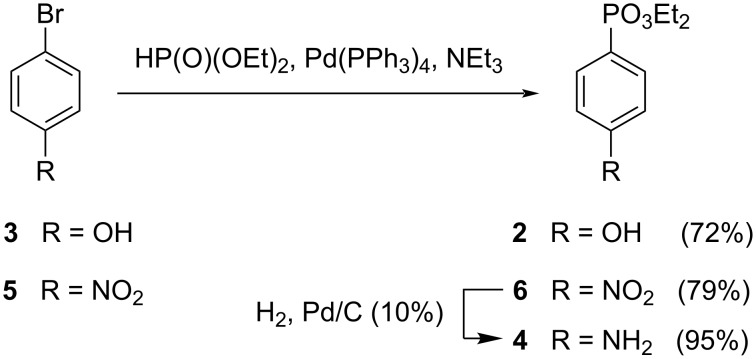
Synthesis of diethyl phenylphosphonates **2, 4** and **6**.

The first nucleophile to be synthesized was diethyl (4-hydroxyphenyl)phosphonate (**2**) [51], starting from 4-bromophenol (**3**) and triethyl phosphite. When this reaction was carried out in the presence of nickel(II) bromide, it afforded compound **2** with a very low yield (2%). When the phosphonation was performed with diethyl phosphonate in the presence of Pd(PPh_3_)_4_ as catalyst and trimethylamine, compound **2** was formed with a good yield (72%) (Scheme 1).

The corresponding (4-nitrophenyl)phosphonate derivative **6** [51] was also prepared, using the same reaction conditions, by the reaction between 1-bromo-4-nitrobenzene (**5**) and diethyl phosphonate, in the presence of Pd(PPh_3_)_4_ as catalyst and triethylamine, since the use of triethyl phosphite in the presence on nickel(II) bromide do not allow the formation of the desired product. Compound **6** [51] was subsequently reduced to the corresponding aniline derivative **4** [52] in the presence of H_2_ and Pd/C (Scheme 1).

**Scheme 2:**
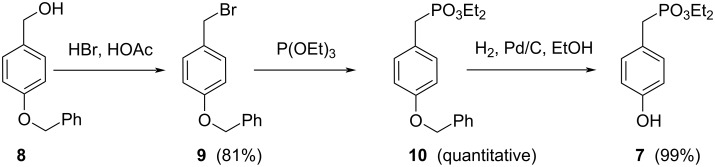
Synthesis of (4-hydroxyphenyl)methylphosphonate **7** starting from [4-(benzyloxy)phenyl]methanol (**8**).

The synthesis of diethyl (4-hydroxyphenyl)methylphosphonate (**7**) [53] started from [4-(benzyloxy)phenyl]methanol (**8**). Compound **8** was submitted to a nucleophilic substitution using hydrobromic acid, as a 33% solution in acetic acid, to afford the corresponding bromide derivative **9** [54] (Scheme 2). Subsequently 1-(benzyloxy)-4-(bromomethyl)benzene (**9**) underwent Michaelis–Arbuzov reaction with triethyl phosphite to afford diethyl [4-(benzyloxy)phenyl]methylphosphonate (**10**). The following hydrogenolysis under H_2_/Pd/C conditions in ethanol, afforded the desired diethyl (4-hydroxyphenyl)methylphosphonate (**7**), in an overall yield of 80%.

**Scheme 3:**
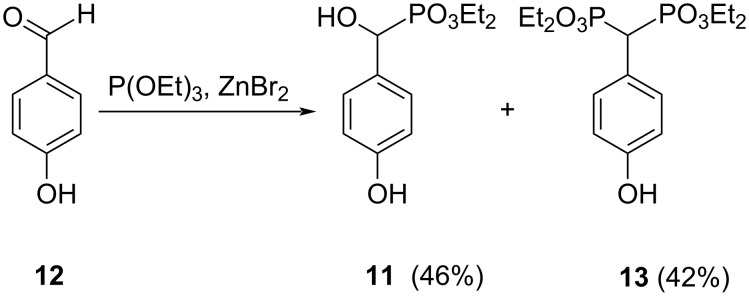
Synthesis of diethyl [hydroxy(4-hydroxyphenyl)methyl]phosphonate (**11**) and tetraethyl [(4-hydroxyphenyl)methylene]bis(phosphonate) (**13**).

The synthesis of diethyl [hydroxy(4-hydroxyphenyl)methyl]phosphonate (**11**) [55] started with the reaction of 4-hydroxybenzaldehyde (**12**) with diethyl phosphonate. Several attempts were carried out under different conditions, using several bases or an acidic resin, but only complex mixtures of products were obtained from which it was not possible to isolate the product. The reaction of 4-hydroxybenzaldehyde (**12**) with triethyl phosphite in the presence of zinc(II) bromide allowed the formation of diethyl [hydroxy(4-hydroxyphenyl)methyl]phosphonate (**11**) and tetraethyl [(4-hydroxyphenyl)methylene]bis(phosphonate) (**13**), which were separated by column chromatography (Scheme 3).

The synthesis of pure tetraethyl [(4-hydroxyphenyl)methylene]bis(phosphonate) (**13**) [56] was successfully achieved, with high yield, by the reaction of diethyl phosphonate with 4-hydroxybenzaldehyde (**12**) in the presence of sodium metal.

**Scheme 4:**
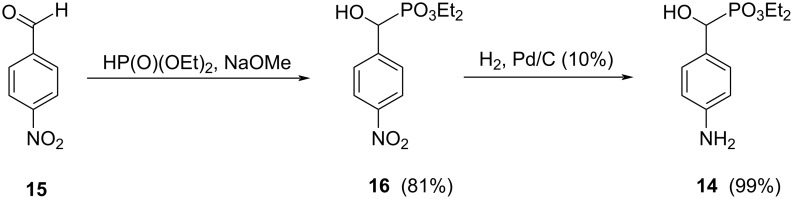
Synthesis of diethyl phenylphosphonates **16** and **14**.

A reaction to obtain the corresponding amino derivative **14** [57] was carried out starting from 4-nitrobenzaldehyde (**15**). Compound **15** reacted with diethyl phosphonate in the presence of a strong base (sodium methoxide) to afford diethyl [hydroxy(4-nitrophenyl)methyl]phosphonate (**16**) in 81% yield (Scheme 4). The hydrogenolysis of this compound under H_2_ on Pd/C afforded quantitatively diethyl [hydroxy(4-aminophenyl)methyl]phosphonate (**14**) (Scheme 4). With the starting compound **15**, the Michaelis-Arbuzov reaction did not afford the tetraethyl bisphosphonate derivative.

**Scheme 5:**
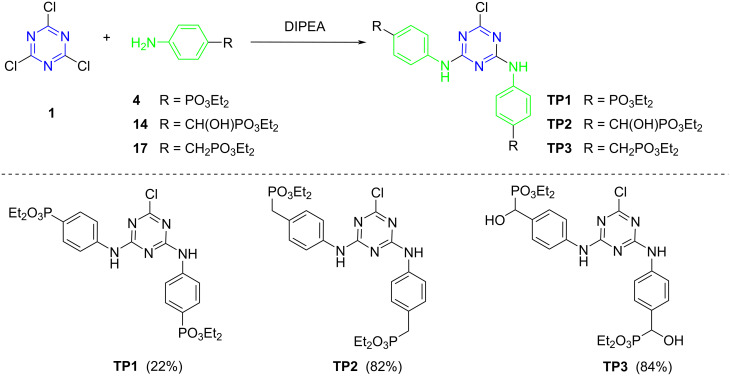
Synthesis of 4-aminophenyltriazinephosphonate derivatives **TP1–TP3**.

To reach the star-shaped *s*-triazine derivatives bearing a phosphonate group, it was considered that the best strategy was to carry out the substitution of chlorine atoms of 2,4,6-trichloro-1,3,5-triazine (cyanuric chloride, **1**) by the previously synthesized nucleophiles. The general scheme to obtain the desired triazinephosphonates (TPs) from the synthesized amino nucleophiles are represented in Scheme 5.

**Figure 2:**
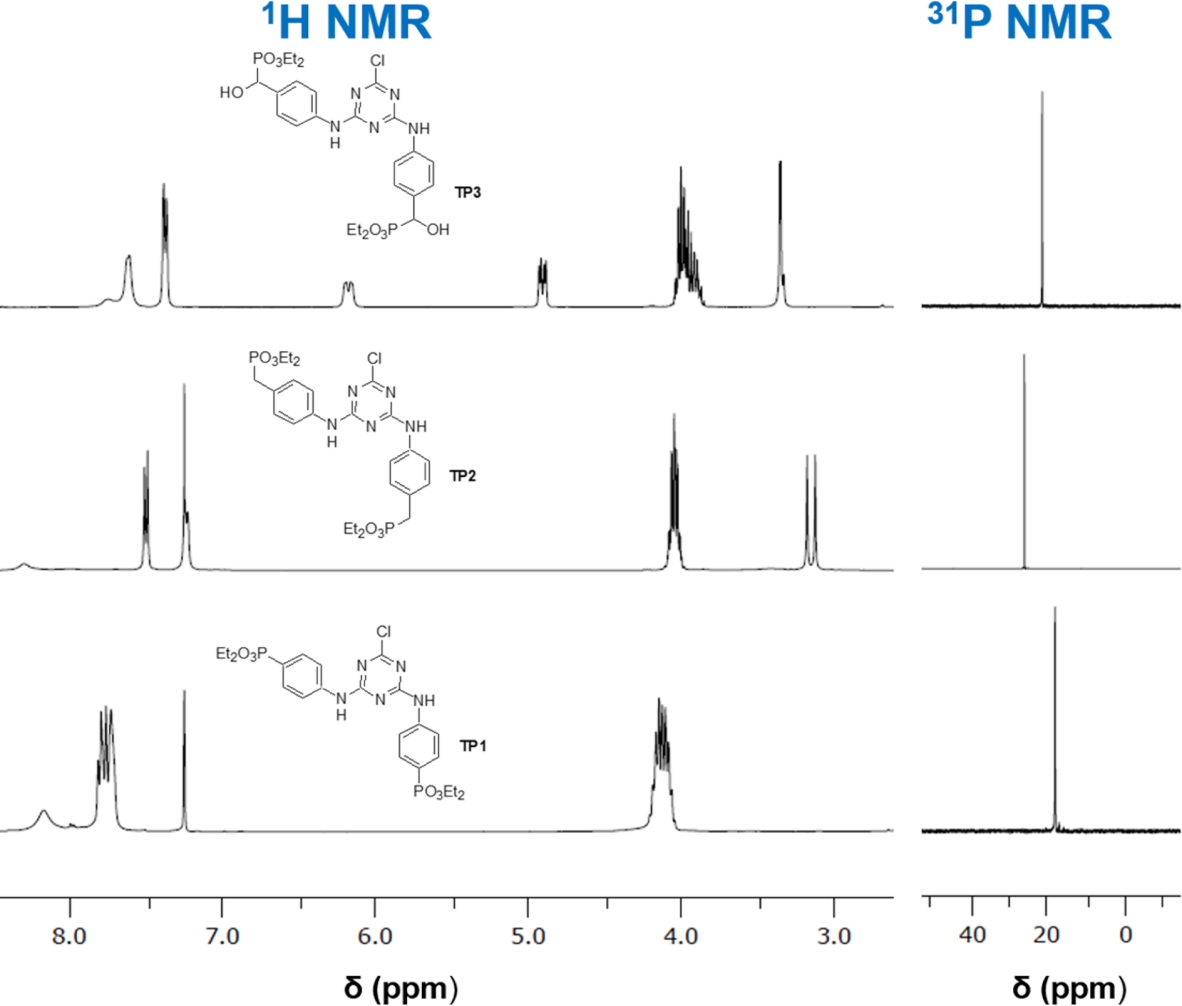
Partial view of ^1^H and ^31^P NMR spectra of 4-aminophenyltriazinephosphonate derivatives **TP1**–**TP3**.

Initially, the 4-aminophenyl derivatives were synthesized in a THF solution, using DIPEA as base. The first reaction was carried out between more than 3 equiv of (4-aminophenyl)phosphonate **4** and cyanuric chloride (**1**). This reaction did not afford the desired trisubstituted triazine; instead, only the disubstituted derivative was achieved (compound **TP1**), in low yield, with a chlorine atom remaining bonded to the triazine ring (Scheme 5). The spectroscopic data of the isolated compound are in accordance with the proposed structure for compound **TP1**. The MS spectrum confirms the presence of a chlorine atom, with a molecular ion MH^+^ and a MH^+^ + 2 peak for ^35^Cl and ^37^Cl isotopes, respectively, with an approximately 3/4 and 1/4 proportion. The symmetry of the obtained compound **TP1** gives simple NMR spectra, with the ^31^P NMR spectrum showing a singlet signal (Figure 2) and the signal at 169.5 ppm, on the ^13^C NMR spectrum, confirming the presence of a chlorine atom bonded to a carbon atom in the proposed structure. Other synthetic attempts were carried out with different reaction conditions, but they usually gave complex mixtures, whose compounds could not be separated, and the trisubstituted triazine derivative was not achieved.

The reaction of cyanuric chloride (**1**) with (4-aminophenyl)methylphosphonate **17** also afforded the disubstituted triazine derivative **TP2** (Scheme 5). Other reactions were carried out at different conditions, but they did not afford the desired trisubstituted derivative. The spectroscopic data are in agreement with the proposed disubstitution pattern, with MS showing MH^+^ and MH^+^ + 2 isotope peaks due to the presence of chlorine in the molecule. In the ^1^H NMR spectrum, the CH_2_P protons appear as a doublet signal at 3.14 ppm, due to the coupling with the ^31^P atom of the phosphonate group (Figure 2). The chemical shift of the ^31^P atoms is observed as a singlet for both phosphorus atoms (Figure 2) and the carbon atom bonded to the chlorine atom is observed at 167.8 ppm.

Also, the reaction with diethyl [hydroxy(4-aminophenyl)methyl]phosphonate (**14**) only afforded the disubstituted derivative **TP3** (Scheme 5), despite the different conditions tested. The spectroscopic data are in agreement with the proposed structure for compound **TP3** (Scheme 5), namely the presence of the chlorine atom in the mass spectrum, due to the presence of isotope peaks, the signal at 168.2 ppm at ^13^C NMR spectrum attributed to the carbon atom bonded to chlorine, and the singlet signal of the phosphorus atom at the ^31^P NMR spectrum.

**Scheme 6:**
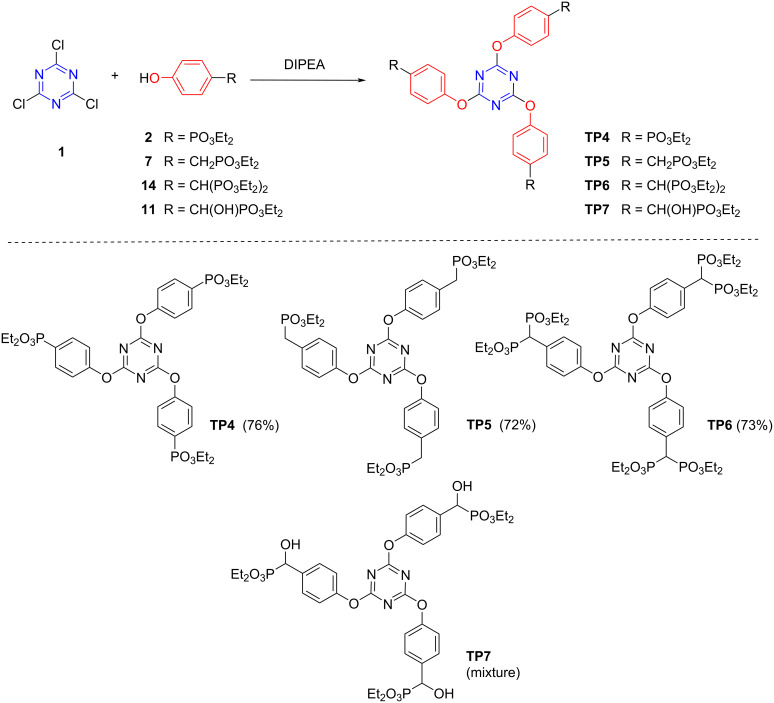
Synthesis of (4-hydroxyphenyl)triazinephosphonate derivatives **TP4**–**TP6**.

**Figure 3:**
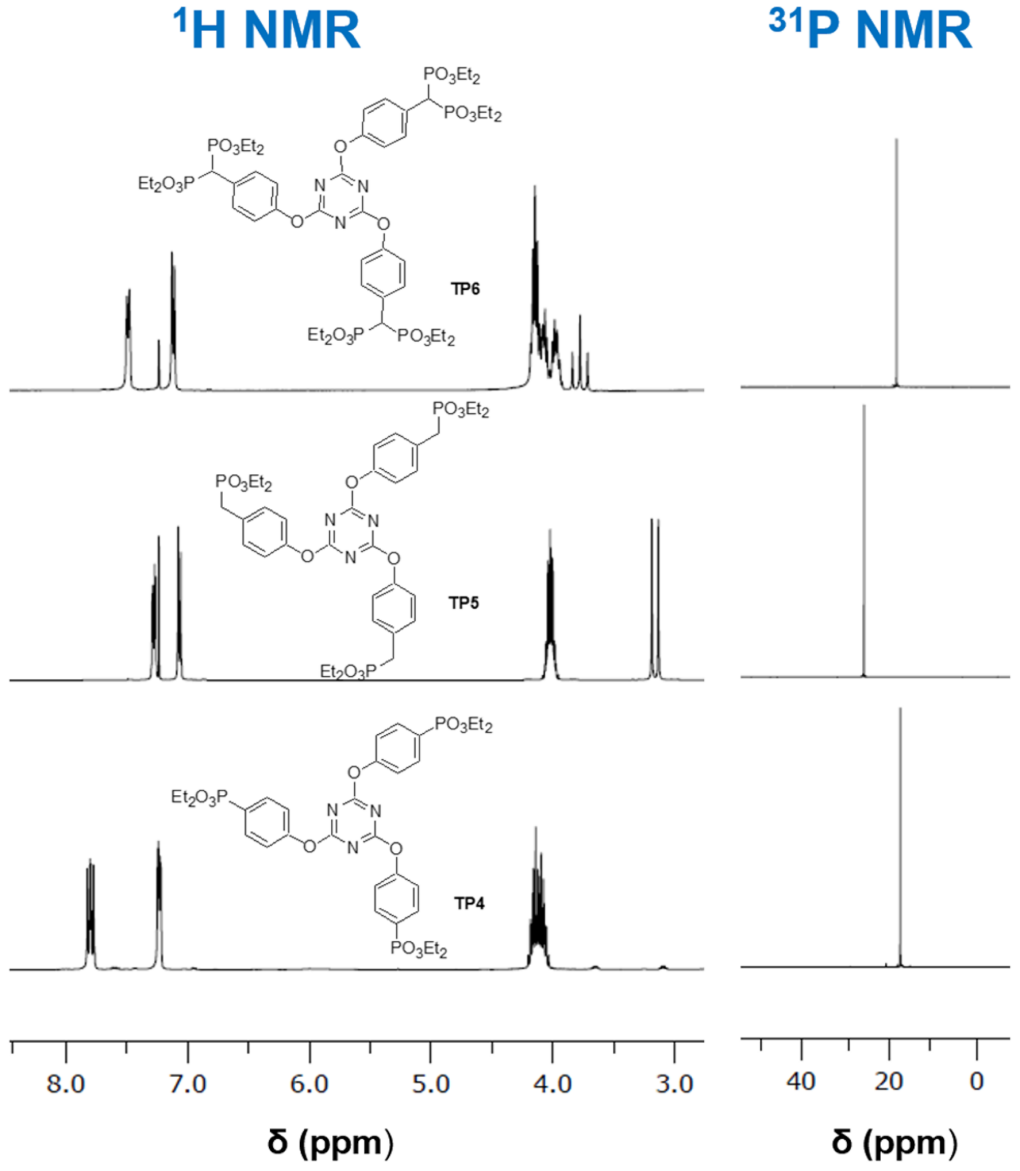
Partial view of ^1^H and ^31^P NMR spectra of (4-hydroxyphenyl)triazinephosphonate derivatives **TP4**–**TP6**.

The synthesis of the corresponding 4-hydroxyphenylphosphonate derivatives followed the same strategy of the amino counterparts’ preparation (Scheme 6). The initial reaction between cyanuric acid (**1**) and diethyl 4-hydroxyphenylphosphonate (**2**) in THF, with DIPEA as base, gave a mixture of products which was purified by column chromatography to afford the desired trisubstituted compound **TP4** (Scheme 6). Since this reaction had a very low yield (17%), another base, Na_2_CO_3_, was used and compound **TP4** was obtained in a good yield (76%). No isotope peaks were observed at the MS of the compound, in agreement with the full displacement of the chlorine atoms. The NMR spectra (Figure 3) presented a similar pattern of the corresponding 4-amino derivative **TP1**. The ^31^P NMR spectrum shows only one singlet in accordance with the symmetry of the molecule, with magnetically equivalent phosphorus atoms.

The synthesis of other 4-hydroxyphenyltriazinephosphonate derivatives **TP5** and **TP6**, starting from diethyl [(4-hydroxyphenyl)methyl]phosphonate (**7**) and tetraethyl [(4-hydroxyphenyl)methylene]bisphosphonate (**13**), were carried out using the same conditions of the previous reaction, including Na_2_CO_3_ as base. These reactions afforded the desired trisubstituted triazine derivatives, compounds **TP5** and **TP6**, respectively, in good yields (>70%) (Scheme 6). The **TP5** was also prepared using DIPEA as a base, but in low yield (25%). All spectra of these compounds are in agreement with the proposed structures (Figure 3).

Several attempts were carried out to prepare triazinephosphonate **TP7** derivative from diethyl [hydroxy(4-hydroxyphenyl)methyl]phosphonate (**11**). However, all the attempts to isolate triazinephosphonate **TP7** derivative failed. The initial reactions between cyanuric acid (**1**) and compound **11**, using Na_2_CO_3_ as base, gave a mixture of products, which were not possible to separate. The data obtained from these mixtures suggest that triazine derivatives with different substitutions patterns are obtained, and that the displacement of the chlorine atom occurred by several substitution degrees by both the oxygen atom of hydroxyphenyl or the hydroxymethyl group of compound **11**, giving a complex mixture. The use of DIPEA as base, under the previous conditions, gave the desired trisubstituted compound **TP7** (crude, 47%) (Scheme 6). However, purification of the crude product by column chromatography led to the decomposition of compound **TP7**.

**Scheme 7:**
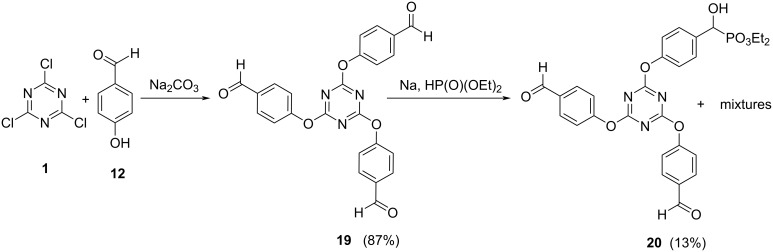
Attempted synthesis of triazinephosphonate **TP7**.

Another strategy was devised to obtain the desired triazinephosphonate **TP7**: The first step was the nucleophilic substitution of the chlorine atoms of triazine **1** by 4-hydroxybenzaldehyde (**12**), followed by the phosphonation of the aldehyde group. To implement this strategy, a reaction between 4-hydroxybenzaldehyde (**12**) and cyanuric chloride (**1**) was performed, in toluene with Na_2_CO_3_ as base, to obtain compound **19** [58] in very good yield (87%) (Scheme 7). Compound **19** was subjected to similar reaction conditions that led to phosphonates **11** and **16**, using diethyl phosphonate in the presence of a base, such as Et_3_N and NaOCH_3_, or even sodium metal, to obtain compound **TP7** (Scheme 7). Unfortunately, these reactions afforded complex mixtures of products and by column chromatography was isolated only compound **20** in low yield (13%). It was not possible to isolate neither di- or triphosphonate derivatives and with this compound it was not possible to prepare a doped membrane (Scheme 7).

### Preparation and proton conduction of doped membranes

Previous studies in our group [26–29] have shown that the incorporation of phosphonic acid derivatives into commercial Nafion polymers increment their proton conduction properties. The synthesized compounds (**TP1**–**TP6**) were attempted to be applied as dopants in the preparation of new proton exchange membranes.

**Figure 4:**
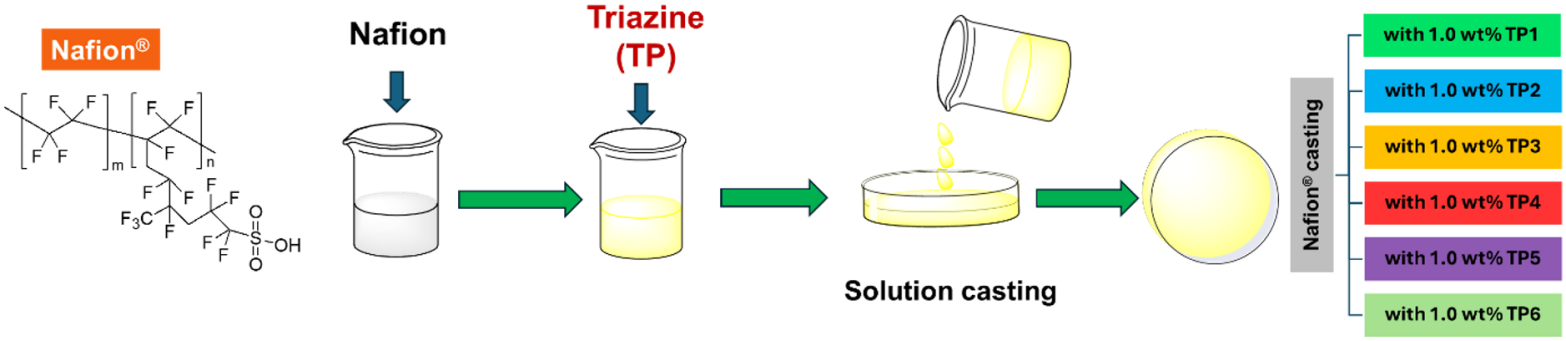
Preparation of the new doped membranes.

With this in mind, new doped membranes were prepared by incorporation of the chosen dopants into a Nafion polymer using a casting method (Figure 4). It was expected that the dopants could act as proton carriers, improving the proton conduction of the membranes. The new membranes had a 1 wt % loading of **TPs** since our previous [27] results showed that 1 wt % is usually the best weight loading for doped Nafion membranes.

The FTIR-ATR spectra of Nafion membranes (Supporting Information File 1, Figure S1) showed the characteristic very strong and broad absorption bands of Nafion near 1200 and 1145 cm^−1^ due to the C–F stretching vibration [59–63]. The phosphonate compounds also present their strongest bands in this region, but they are overlapped by the more intense Nafion bonds due to the small wt % loading of the dopants. As a result, despite the visible slight dark-brown color, observed by a visual inspection of the membranes, only discrete changes are observed in the spectra of new membranes compared to commercial Nafion [64]. Other characteristic bands of S–O group, CF_2_–CF and C–O–C are observed at near 1050, 980 and 960 cm^−1^, respectively, in the FTIR spectra [63].

**Figure 5:**
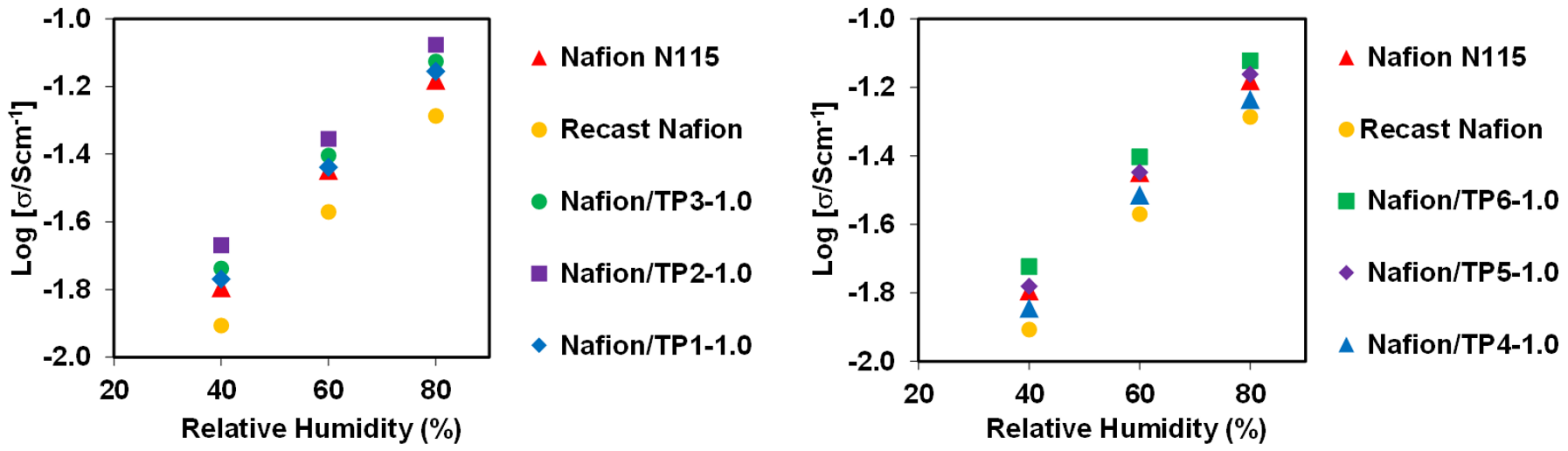
Comparison of in-plane proton conductivity vs RH of Nafion doped membranes, at 60 °C.

The proton conductivity of the new proton exchange membrane is a key property relevant, for example, to the performance of fuel cells. The study of the protonic conductivities of the new modified membranes were determined, in-plane, at 60 ºC, in different relative humidity (RH) conditions (40, 60 and 80%), through electrochemical impedance spectroscopy (EIS) (Figure 5). A prepared recast Nafion and a commercial Nafion N115 membrane were also submitted to EIS analysis, under the same experimental conditions of the prepared membranes, for comparison of their proton conductivities.

An increase in the proton conductivity of all membranes with increasing of RH was observed. All membranes showed a near 4-fold increment in their proton conductivities between 40% to 80% RH conditions.

All membranes showed a better proton conductivity than the recast Nafion membrane. Also, all prepared membranes, with exception of the membrane doped with **TP4**, showed better proton conductivity than commercial Nafion N115.

It was also observed that the membranes doped with aminophenylphosphonate derivatives (**TP1**, **TP2** and **TP3**) show slightly higher proton conductivities compared with hydroxyphenyl derivatives (**TP4**, **TP5** and **TP6**). In both series (aminophenylphosphonate or hydroxyphenylphosphonate derivatives), the membranes doped with phenylphosphonates (**TP1** and **TP4**) showed the lowest proton conductivity compared with the membranes doped with other amino- or hydroxy derivatives. In the case of the membrane doped with triazine **TP6**, with a methylbisphosphonate structure, it is observed similar results compared to the membrane doped with **TP3** with a hydroxymethylphosphonate structure. The best value was observed for the Nafion membrane doped with compound **TP2**, at 80% RH, with a proton conductivity of 83.8 mS·cm^–1^.

## Conclusion

This study presents the synthesis of several triazinephosphonates with 4-aminophenyl or 4-hydroxyphenyl moieties, used as dopants in the preparation of new Nafion membranes. The synthesis of these triazinephosphonate derivatives were achieved through the substitution of chlorine atoms of cyanuric chloride (**1**). In these syntheses, different bases were used with different results, with Na_2_CO_3_ being used to obtain 4-hydroxyphenyl derivatives **TP4**, **TP5** and **TP6**, while DIPEA was used to prepare the 4-aminophenyl counterparts and **TP7**. In the case of the 4-aminophenyl derivatives, only the disubstituted triazine compounds **TP1**–**TP3** were isolated. The proposed structures for these compounds were supported by the spectroscopic data, including NMR, FTIR and MS spectra.

New doped Nafion membranes were prepared, with a 1 wt % loading of the dopant, by a casting method developed previously in our group. The proton conduction properties of the new membranes were evaluated by EIS, under different RH conditions, at 60 ºC. The EIS results of the new membranes endorse the proposed strategy delivering new membranes with a better proton conductivity. These results showed that the incorporation of the dopants promotes an increase in the proton conduction of the new membranes, with higher proton conductivity values than recast Nafion and commercial Nafion N115 (with the exception of the membrane doped with **TP4**) under the same experimental conditions. The RH marks a strong influence with large increments in the proton conductivity of all membranes with the increase of RH, up to 4.2-fold, when the measurements were done at 80% RH compared with 40% RH results. Also, disubstituted (4-aminophenyl)triazinephosphonate-doped membranes showed better proton conductivities than trisubstituted (4-hydroxyphenyl)triazinephosphonate-doped membranes in the same experimental conditions. The highest proton conductivity was observed for Nafion doped membrane with compound **TP2**, with a 1.0 wt % loading, with 84 mS·cm^−1^.

## Experimental

### Materials and methods

Cyanuric chloride (**1**) and diethyl (4-aminophenyl)methylphosphonate (**17**) are commercially available (Sigma-Aldrich, Alfa Aesar). Other acquired reagents and deuterated solvents were used as received, without further purification. Solvents and air-sensitive reagents were distilled under a dry nitrogen atmosphere. Dry THF was distilled from sodium benzophenone ketyl.

A Nafion N115 film was acquired from FuelCell Store and a 20 wt % mixture in lower aliphatic alcohols and water (34%) of Nafion perfluorinated resin solution was purchased from Sigma-Aldrich.

Purification of reaction products was done by column chromatography on silica gel (230–400 mesh) with the appropriate eluent mixture and using a positive pressure of nitrogen.

### Spectroscopic characterization

The characterization of the dopants was carried out by Fourier-transform infrared spectroscopy (FTIR), nuclear magnetic resonance (NMR) spectroscopy and mass spectrometry (MS). ^1^H, ^13^C and ^31^P NMR characterization was done using different one- and two-dimensional techniques, and were obtained on a Bruker Avance III HD 400 (^1^H 400 MHz, ^13^C NMR 100 MHz, ^31^P 162 MHz) spectrometer, with the chemical shifts (δ) indicated in ppm, and coupling constants (*J*) in Hz.

The FTIR characterization of the dopants was done on a PerkinElmer FT-IR Spectrum BX Fourier Transform spectrometer, using KBr discs, and the characterization of the membranes was carried out on a Perkin Elmer Spectrum Two, with an attenuated total reflectance (ATR) module, with a wavenumber range from 450 to 4000 cm^−1^, and their band wavelengths are quoted in cm^−1^.

Low-resolution and high-resolution (HRMS) mass spectra (MS) were performed on an APEX-Q (Bruker Daltonics) instrument at ‘C.A.C.T.I. - Unidad de Espectrometria de Masas’, at the University of Vigo, Spain. Melting points were determined on a Reichert Thermovar melting point apparatus and are uncorrected.

### Proton conductivity

In-plane proton conductivity (σ) evaluation of the new membranes was performed by electrochemical impedance spectroscopy (EIS), on a commercial BT-112 BekkTech conductivity cell (Scribner Associates Inc.), with a frequency response analyzer Solartron 1250, coupled to a Solartron 1286 electrochemical interface. The measurements were performed with a test signal amplitude of 10 mV, over a frequency range of 65 kHz to 5 Hz. The bulk resistance (*R_b_*) of the membranes were calculated using the ZView software (Version 2.6b, Scribner Associates). A Binder KBF 115 climatic chamber was used to perform the measurements at a temperature of 60 °C and different relative humidity (RH) conditions (40, 60 and 80%). The measurements were performed directly from the temperature-controlled humidity chamber, after a 2 h equilibration period.

The proton conductivity (σ) was calculated using Equation 1


[1] $$\sigma =\frac{L}{AR_{b}},$$

where *L* – distance between the two electrodes (cm), *R_b_* – bulk resistance (Ω), and *A* - cross-sectional area (cm^2^).

### Preparation of the dopants

The preparation of all compounds is described in Supporting Information File 1.

### Membrane preparation

Membranes were prepared by a casting method using Nafion^®^/DMAc solutions, based on our previous works, using 1 wt % loading of **TP** dopants. The 20 wt % Nafion solution was dried under reduced pressure, at 40 °C, until a dry residue was obtained. A new 10 wt % solution of Nafion was obtained by dissolution of the dried Nafion in the required amount of *N,N*-dimethylacetamide (DMAc). The **TP** dopant quantity was added to DMAC solution and the mixture was stirred during 1–5 h, in an ultrasonic bath, to guarantee the complete dissolution of dopants. The resulting solutions were casted on a 5 cm diameter Petri dish and slowly evaporated, until obtaining homogeneous membranes. The resulting membranes were dried in a vacuum oven at 60 °C, and were followed by their annellation for 2 h, at 140 °C. The membranes were activated by a sequential treatment, with 1 h for each step, by boiling them in H_2_O_2_ solution (3%), washing with hot deionized water, boiling in a 0.5 M sulfonic acid solution, and washing again with hot deionized water. After activation, the membranes were kept in deionized water until their use. The new membranes were labelled as Nafion/TP*i*-1.0, respectively, where *i* indicates the specific triazine used, and 1.0 specifies the wt % of dopant. Recast Nafion films were also prepared, for comparison, without the incorporation of TPs.

## Supporting Information

File 1 Experimental data.

## Data Availability

All data that supports the findings of this study is available in the published article and/or the supporting information to this article.
