# Supplementary material for: New triazinephosphonate dopants for Nafion proton exchange membranes (PEM)
Source: Beilstein J Org Chem. 2024 Jul 17;20:1623–34. doi: 10.3762/bjoc.20.145 (PMC11285047; doi:10.3762/bjoc.20.145)
Supplement: File 1 — Experimental data. [file Beilstein_J_Org_Chem-20-1623-s001.pdf]

BEILSTEIN JOURNAL OF ORGANIC CHEMISTRY

Supporting Information

for

New triazinephosphonate dopants for Nafion proton exchange membranes (PEM)

Fátima C. Teixeira, António P. S. Teixeira and C. M. Rangel

Beilstein J. Org. Chem. **2024,** *20,* 1623–1634. doi:10.3762/bjoc.20.145

Experimental data

License and Terms: This is a supporting information file under the terms of the Creative Commons Attribution License (https://creativecommons.org/licenses/by/4.0). Please note that the reuse, redistribution and reproduction in particular requires that the author(s) and source are credited and that individual graphics may be subject to special legal provisions.
The license is subject to the *Beilstein Journal of Organic Chemistry* terms and conditions: (https://www.beilstein-journals.org/bjoc/terms)

Table of Contents

1. Experimental...S1

1.1. Materials and Methods..S1

1.2. Spectroscopic Characterization...S1

1.3. Proton conductivity..S2

1.4. Preparation of the dopants...S3

1.5. Membrane Preparation S15

1.6. FTIR-ATR spectra of the new membranes S16

2. References S16

1. Experimental

1.1. Materials and Methods

Cyanuric chloride (**1**) and diethyl (4-aminophenyl)methylphosphonate (**17**) are commercially available (Sigma-Aldrich, Alfa Aesar). Other acquired reagents and deuterated solvents were used as received, without further purification. Solvents and air-sensitive reagents were distilled under a dry nitrogen atmosphere. Dry THF was distilled from sodium benzophenone ketyl.

A Nafion N115 film was acquired from FuelCell Store and a 20 wt % mixture in lower aliphatic alcohols and water (34%) of Nafion perfluorinated resin solution was purchased from Sigma Aldrich.

Purification of reaction products was done by column chromatography on silica gel (230–400 mesh) with the appropriate eluent mixture and using a positive pressure of nitrogen.

1.2. Spectroscopic characterization

The characterization of the dopants was carried out by Fourier-transform infrared spectroscopy (FTIR), nuclear magnetic resonance (NMR) spectroscopy and mass spectra spectrometry (MS). ^{1}H, ^{13}C and ^{31}P NMR characterization was done using different one- and two-dimensional techniques, and were

obtained on a Bruker Avance III HD 400 (^{1}H 400 MHz, ^{13}C NMR 100 MHz, ^{31}P 162 MHz) spectrometers, with the chemical shifts (δ) indicated in ppm, and coupling constants (*J*) in Hz.

The FTIR characterization of the dopants was done on a PerkinElmer FT-IR Spectrum BX Fourier Transform spectrometer, using KBr discs, and the characterization of the membranes was carried out on a Perkin Elmer Spectrum Two, with an attenuated total reflectance (ATR) module, with a wavenumber range from 450 to 4000 cm^{-1}, and their band wavelengths are quoted in cm^{-1}.

Low-resolution and high-resolution (HRMS) mass spectra (MS) were performed on an APEX-Q (Bruker Daltonics) instrument at 'C.A.C.T.I. - Unidad de Espectrometria de Masas', at University of Vigo, Spain.

Melting points were determined on a Reichert Thermovar melting point apparatus and are uncorrected.

1.3. Proton conductivity

In-plane proton conductivity (σ) evaluation of the new membranes was performed by electrochemical impedance spectroscopy (EIS), on a commercial BT-112 BekkTech conductivity cell (Scribner Associates Inc.), with a frequency response analyzer Solartron 1250, coupled to a Solartron 1286 electrochemical interface. The measurements were performed with a test signal amplitude of 10 mV, over a frequency range of 65 kHz to 5 Hz. The bulk resistance (R_b) of the membranes were calculated using the ZView software (Version2.6b, Scribner Associates). A Binder KBF 115 climatic chamber was used to perform the measurements at a temperature of 60 °C and different relative humidity (RH) conditions (40, 60 and 80%). The measurements were performed directly from the temperature-controlled humidity chamber, after a 2 h equilibration period.

The proton conductivity (σ) was calculated using the equation (1)

$$\sigma = \frac{L}{AR_b} \qquad (1)$$

where L - distance between the two electrodes (cm), R_b – bulk resistance (Ω), and A - cross-sectional area (cm^2).

1.4. Preparation of the dopants

Synthesis of diethyl 4-hydroxyphenylphosphonate (2)

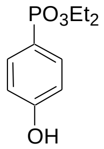

To a solution of 4-bromophenol (**3**, 400 mg, 2.31 mmol) in EtOH (25 mL) was added diethyl phosphonate (0.45 mL, 3.48 mmol), triethylamine (0.65 mL, 4.62 mmol) and $Pd(PPh_3)_4$ (134 mg, 0.116 mmol, 5%). The reaction mixture was heated to reflux and stirred under N_2 for 24 h. After cooling to room temperature, the solvent was removed under reduced pressure, the residue was dissolved in EtOAc and extracted with 10% aqueous NaOH solution. The aqueous phase was washed with EtOAc, acidified with 10% aqueous HCl solution and extracted with EtOAc. The combined organic extracts were dried over anhydrous $MgSO_4$. After filtration, the solvent was removed under reduced pressure. The resulting oil was purified by column chromatography (1:1 EtOAc:acetone) to give compound **2** [1] (383 mg, 72%) as an oil.^{1}H NMR (400 MHz, $CDCl_3$): δ (ppm) = 1.31 (t, *J* = 7.2, 6H, 2x OCH_2CH_3), 4.04-4.13 (m, 4H, 2x OCH_2CH_3), 6.98 (dd, *J* = 8.4 and *J* = 3.6, 2H, Ar*H*), 7.64 (dd, *J* = 13.2 and *J* = 8.4, 2H, Ar*H*). ^{31}P NMR (162 MHz, H_3PO_4/ $CDCl_3$): δ (ppm) = 20.6.

Synthesis of diethyl 4-nitrophenylphosphonate (6)

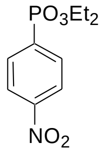

To a solution of 1-bromo-4-nitrobenzene (**5**, 500 mg, 2.48 mmol) in EtOH (25 mL) was added diethyl phosphonate (0.48 mL, 3.72 mmol), triethylamine (0.69 mL, 4.96 mmol) and $Pd(PPh_3)_4$ (150 mg, 0.124 mmol, 5%). The reaction mixture was heated to reflux and stirred under N_2 for 24 h. After cooling to room temperature, the solvent was removed under reduced pressure. The resulting oil was purified by column chromatography (EtOAc) to give compound **6** [1] (511 mg, 79%) as a solid. ^{1}H NMR (400 MHz, $CDCl_3$): δ (ppm) = 1.33 (t, *J* = 7.2, 6H, 2x OCH_2CH_3), 4.09-4.20 (m, 4H, 2x OCH_2CH_3), 7.99 (dd, *J* = 12.6 and *J* = 8.4, 2H, Ar*H*), 8.29 (dd, *J* = 8.4 and *J* = 3.2, 2H, Ar*H*). ^{31}P NMR (162 MHz, H_3PO_4/ $CDCl_3$): δ (ppm) = 15.0.

Synthesis of diethyl (4-aminophenyl)phosphonate (4)

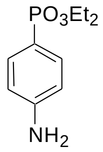

To a solution of compound **6** (350 mg, 1.35 mmol) in EtOH (10 mL) was added 10% palladium on activated carbon (70 mg, 20 w/w %) and the solution was stirred at room temperature under H_2 atmosphere during 2 h. The reaction mixture was filtered through a plug of Celite®, washed through with EtOH and concentrated *in vacuo* to yield compound **4** [2] as a yellow solid (293 mg, 95%). ^{1}H NMR (400 MHz, $CDCl_3$): δ (ppm) = 1.30 (t, *J* = 7.2, 6H, 2x OCH_2CH_3), 4.02-4.13 (m, 4H, 2x OCH_2CH_3), 6.92 (d, *J* = 5.2, 2H, Ar*H*), 7.63 (dd, *J* = 12.6 and *J* = 8.4, 2H, Ar*H*). ^{31}P NMR (162 MHz, H_3PO_4/ $CDCl_3$): δ (ppm) = 20.0.

Synthesis of 1-(benzyloxy)-4-(bromomethyl)benzene (9)

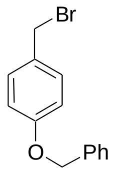

A solution of [4-(benzyloxy)phenyl]methanol (**8**, 2.40 g, 11.20 mmol) in acetic acid (7.5 mL) was cooled to 0 ºC, and a solution of hydrobromic acid (7.2 mL, 33% in acetic acid) was added to the solution. The mixture was warmed to room temperature during 30 min. The reaction mixture was added to ice-water, and the white solid was filtered and washed with water. The white solid was redissolved in EtOAc, washed with brine and dried over anhydrous $MgSO_4$. After filtration, the solvent was removed under reduced pressure to afford compound **9** [3] (2.53 g, 81%) as a white solid. ^{1}H NMR (400 MHz, $CDCl_3$): δ (ppm) = 4.50 (s, 2H, CH_2), 5.07 (s, 2H, OCH_2), 6.93-6,95 (m, 2H, Ar*H*), 7.32-7.44 (m, 7H, Ar*H*).

Synthesis of diethyl [4-(benzyloxy)phenyl]methylphosphonate (10)

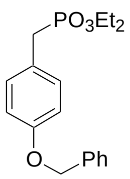

1-(Benzyloxy)-4-(bromomethyl)benzene (**9**, 1.02 g, 3.68 mmol) and triethyl phosphite (1 mL, 5.83 mmol) in a DMF solution (3 mL), under a nitrogen atmosphere, were heated at 155 °C for 3 h. Upon cooling, water was added to the mixture. The resulting aqueous layer was extracted with EtOAc and the combined organic extract was dried over anhydrous $MgSO_4$ and filtered. The solution was dried in a vacuum rotary evaporator to afford an oil that, after being purified by column chromatography (2:3 acetone/petroleum ether), gave compound **10** [4] as colorless oil, in quantitatively yield. ^{1}H NMR (400 MHz, $CDCl_3$): δ (ppm) = 1.28 (t, *J* = 7.2, 6H, 2x OCH_2CH_3), 3.09 (d, *J* = 21.2, 2H, C*H*$_2$P), 3.95-4.05 (m, 4H, 2x OC*H*$_2$CH$_3$), 5.04 (s, 2H, OC*H*$_2$Ar), 6.92 (d, *J* = 8.4, 2H, Ar*H*), 7.21 (dd, *J* =8.8 and *J* =2.4, 2H, Ar*H*), 7.30-7.44 (m, 5H, Ar*H*). ^{31}P NMR (162 MHz, H_3PO_4/ $CDCl_3$): δ (ppm) = 26.9.

Synthesis of diethyl 4-hydroxybenzylphosphonate (7)

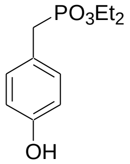

To a solution of compound **10** (1.28 g, 3.83 mmol) in EtOH (25 mL) was added 10% palladium on activated carbon (256 mg, 20 w/w %) and the solution was stirred at room temperature under H_2 atmosphere for 24 h. The reaction mixture was filtered through a plug of Celite®, rinsed with EtOH and concentrated in vacuo to yield compound **7** [4] as a white solid (930 mg, 99%). ^{1}H NMR (400 MHz, $CDCl_3$): δ (ppm) = 1.26 (t, *J* = 7.2, 6H, 2x OCH$_2$C*H*$_3$), 3.06 (d, *J* = 20.8, 2H, C*H*$_2$P), 3.97-4.07 (m, 4H, 2x OC*H*$_2$CH$_3$), 6.14 (br s, 1H, O*H*), 6.64 (d, *J* = 8.0, 2H, Ar*H*), 7.03 (dd, *J* = 8.4 and *J* = 2.8, 2H, Ar*H*). ^{31}P NMR (162 MHz, H_3PO_4/ $CDCl_3$): δ (ppm) = 27.6.

Synthesis of diethyl [hydroxy(4-hydroxyphenyl)methyl]phosphonate (11) and tetraethyl [(4-hydroxyphenyl)methylene]bisphosphonate (13)

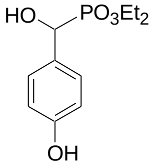

11

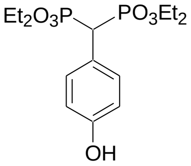

13

To a solution of 4-hydroxybenzaldehyde **12** (337 mg, 2.759 mmol) and triethyl phosphite (1.4 mL, 8.278 mmol), was added $ZnBr_2$ (62 mg, 0.276 mmol) and the mixture was stirred overnight, under N_2, at room

temperature. The reaction mixture was poured into an ice aqueous HCl (1 M) solution and the obtained aqueous layer was extracted with EtOAc. The combined organic extracts were dried over anhydrous $MgSO_4$, filtered and the solvent removed under vacuum. The resulting oil was purified by column chromatography (1:1 ethyl acetate/acetone) to give compound **11** [5] (328 mg, 46%) and compound **13** [6] (434 mg, 42%), as white solids.

Compound **11**: ^1H NMR (400 MHz, DMSO): δ (ppm) = 1.12 (t, *J* = 7.2, 3H, OCH_2CH_3), 1.18 (t, *J* = 7.2, 3H, OCH_2CH_3), 3.79 -4.00 (m, 4H, 2x OCH_2CH_3), 4.78 (dd, *J* = 12.0 and *J* = 5.6, 1H, C*H*P(OH)), 5.97 (dd, *J* = 16.0 and *J* = 5.6, 1H, CHP(O*H*)), 6.72 (d, *J* = 8.0, 2H, Ar*H*), 7.22 (dd, *J* = 8.4 and *J* = 2.0, 2H, Ar*H*), 9.38 (s, 1H, ArO*H*). ^{31}P NMR (162 MHz, H_3PO_4/ DMSO): δ (ppm) = 22.3.

Compound **13**: ^1H NMR (400 MHz, $CDCl_3$): δ (ppm) = 1.13 (t, *J* = 7.0, 6H, 2x OCH_2CH_3), 1.28 (t, *J* = 7.0, 6H, 2x OCH_2CH_3), 3.63 (t, *J* = 25.4, 1H, P_2CH), 3.87 (m, 2H, OCH_2CH_3), 4.01 (m, 2H, OCH_2CH_3), 4.11 (m, 4H, 2x OCH_2CH_3), 6.68 (d, *J* = 8.4, 2H, Ar*H*), 7.20 (d, *J* = 8.8, 2H, Ar*H*). ^{31}P NMR (162 MHz, H_3PO_4/ DMSO): δ (ppm) = 19.0.

Synthesis of tetraethyl [(4-hydroxyphenyl)methylene]bisphosphonate (13)

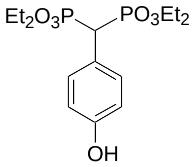

13

Small portions of sodium metal (0.3 g, 13 mmol) were cautiously added to diethyl phosphonate (5 mL) at room temperature. Compound **12** (122 mg, 1.0 mmol) was carefully added to this solution and the reaction mixture was stirred at room temperature for 48 h, after which it was quenched with water (100 mL). The aqueous layer was extracted with $CHCl_3$ (3x 50 mL). The $CHCl_3$ solution was concentrated under reduced pressure and the obtained residue was washed with hexane and dried *in vacuo*. The resulting oil was purified by column chromatography (1:1 EtOAc:Acetone) to give compound **13** [6] (326 mg, 86%) as colourless crystals.

Synthesis of diethyl (4-nitrophenyl)(hydroxy)methylphosphonate (16)

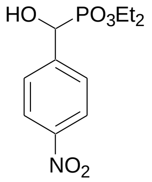

In a manner similar to [7], diethyl phosphonate (2.1 mL) was added to a suspension of NaOMe (1.530 g, 82.65 mmol) in DCM (25 mL) at 0 ºC. The reaction mixture was stirred at 0 ºC, under N_2, during 1 h, and 4-nitrobenzaldehyde (**15**, 2.00 g, 13.23 mmol) was added. The mixture was stirred overnight at room temperature. The reaction mixture was then quenched by addition of aqueous HCl solution (0.1 M) and the aqueous layer was extracted with EtOAc. The combined organic extracts were washed with brine and dried over anhydrous $MgSO_4$. After filtration, the solvent was removed under reduced pressure, followed by recrystallization from Et_2O to give compound **16** [8] as a white solid (3.10 g, 81%). ^{1}H NMR (400 MHz, $CDCl_3$): δ (ppm) = 1.27 (t, *J* = 7.2, 3H, OCH_2CH_3), 1.30 (t, *J* = 7.2, 3H, OCH_2CH_3), 4.07-4.15 (m, 4H, 2x OCH_2CH_3), 5.18 (d, *J* = 12.0, 1H, C*H*P(OH)), 7.68 (d, *J* = 7.2, 2H, Ar*H*), 8.23 (d, *J* = 8.8, 2H, Ar*H*). ^{31}P NMR (162 MHz, H_3PO_4/ $CDCl_3$): δ (ppm) = 19.7. ^{1}H NMR (400 MHz, DMSO): δ (ppm) = 1.67 (t, *J* = 7.2, 3H, OCH_2CH_3), 1.78 (t, *J* = 7.2, 3H, OCH_2CH_3), 3.91 -4.06 (m, 4H, 2x OCH_2CH_3), 5.20 (dd, *J* = 15.6 and *J* = 5.6, 1H, C*H*P(OH)), 6.53 (dd, *J* = 14.6 and *J* = 5.6, 1H, CHP(O*H*)), 7.69 (dd, *J* = 8.8 and *J* = 2.0, 2H, Ar*H*), 8.22 (d, *J* = 8.4, 2H, Ar*H*). ^{31}P NMR (162 MHz, H_3PO_4/ DMSO): δ (ppm) = 20.3.

Synthesis of diethyl (4-aminophenyl)(hydroxy)methylphosphonate (14)

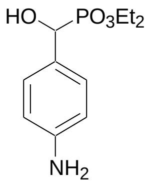

To a solution of compound **16** (384 mg, 1.328 mmol) in EtOH (10 mL) was added 10% palladium on activated carbon (77 mg, 20 w/w %) and the solution was stirred at room temperature under H_2 during 3 h. The reaction mixture was filtered through a plug of Celite®, washed through with EtOH and concentrated *in vacuo* to yield compound **14** [8] as a pale yellow solid (341 mg, 99%).^{1}H NMR (400 MHz, DMSO): δ (ppm) = 1.10 (t, *J* = 7.2, 3H, OCH_2CH_3), 1.18 (t, *J* = 7.2, 3H, OCH_2CH_3), 3.74 -3.98 (m, 4H, 2x OCH_2CH_3), 4.66 (dd, *J* = 11.6 and *J* = 5.2, 1H, C*H*P(OH)), 5.06 (br s, 2H, $ArNH_2$), 5.82 (dd, *J* =

16.4 and J = 5.6, 1H, CHP(OH)), 6.50 (d, J = 8.0, 2H, ArH), 7.06 (d, J = 8.0, 2H, ArH). ^{31}P NMR (162 MHz, H_3PO_4/ DMSO): δ (ppm) = 22.7.

Synthesis of tetraethyl {[(6-chloro-1,3,5-triazine-2,4-diyl)bis(azanediyl)]bis(4,1-phenylene)}bis(phosphonate) (TP1)

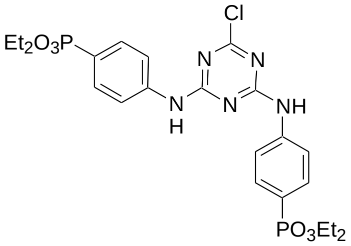

To a solution of diethyl (4-aminophenyl)phosphonate (**4**, 272 mg, 1.186 mmol, 3.3 equiv) in THF (10 mL), under N_2, was added cyanuric chloride (**1**, 65 mg, 0.351 mmol) and DIPEA (0.6 mL, 3.580 mmol). The mixture was heated to reflux and stirred under N_2 overnight. The reaction mixture was cooled to room temperature and the solvent was removed in vacuum. The resulting oil was purified by column chromatography (1:1 ethyl acetate/acetone) to give compound **TP1** (58 mg, 22%) as a white solid. ν_{max} (KBr) (cm^{-1}): 3266, 3132, 3049, 2982, 2931, 2908, 1616, 1603, 1568, 1548, 1489, 1451, 1407, 1371, 1318, 1296, 1277, 1224, 1190, 1164, 1135, 1097, 1053, 1026, 986, 962, 838, 801, 761, 686, 667, 561, 538, 464. ^{1}H NMR (400 MHz, $CDCl_3$): δ (ppm) = 1.33 (t, J = 7.2, 12H, 4x OCH$_2$CH_3), 4.06-4.19 (m, 8H, 4x OCH_2CH$_3$), 7.74-7.83 (m, 8H, ArH), 8.18 (brs, 3H, NH). ^{13}C NMR (100 MHz, $CDCl_3$): δ (ppm) = 16.5 (d, J_{CP} = 6.5, CH$_3$), 62.4 (d, J_{CP} = 5.3, OCH$_2$), 120.4 (d, J_{CP} = 12.6, Ar:C), 123.5 (d, J_{CP} = 190.4, Ar:C), 133.0 (d, J_{CP} = 10.8, Ar:C), 141.4 (Ar:C), 163.9 (C Triazine), 169.5 (C-Cl Triazine).^{1}P NMR (162 MHz, H_3PO_4/ $CDCl_3$): δ (ppm) = 18.4. MS (ESI): m/z = 572 (MH$^+$+2, 30%), 570 (MH$^+$, 100%). HRMS (ESI) m/z calcd for $C_{23}H_{31}ClN_5O_6P_2$ 570.14326 (^{35}Cl isotope) [MH]$^+$, found 570.14199.

Synthesis of tetraethyl ({[(6-chloro-1,3,5-triazine-2,4-diyl)bis(azanediyl)]bis(4,1-phenylene)}bis(methylene))bis(phosphonate) (TP2)

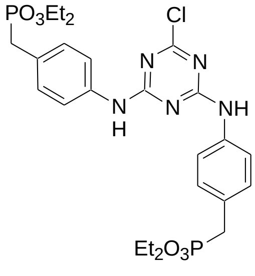

To a solution of diethyl (4-aminophenyl)methylphosphonate (**17**, 200 mg, 0.822 mmol, 3.4 equiv) in THF (10 mL), under N_2, was added cyanuric chloride (**1**, 45 mg, 0.243 mmol) and DIPEA (0.43 mL, 2.479 mmol). The mixture was heated to reflux and stirred under N_2 overnight. The reaction mixture was cooled to room temperature and the solvent was removed in vacuum. The resulting oil was purified by column chromatography (1:1 ethyl acetate/acetone) to give compound **TP2** as a white solid (159 mg, 82%). mp 211-212 ^{o}C. ν_{max} (KBr) (cm^{-1}): 3254, 3191, 3165, 3084, 2981, 2930, 2908, 1624, 1574, 1506, 1436, 1420, 1396, 1321, 1301, 1268, 1227, 1190, 1164, 1099, 1056, 1023, 983, 970, 845, 804, 766, 713, 690, 591, 546, 537, 509. ^{1}H NMR (400 MHz, $CDCl_3$): δ (ppm) = 1.25 (t, *J* = 7.2, 12H, 4x OCH_2CH_3), 3.14 (d, *J* = 21.6, 4H, 2x CH_2P), 4.00-4.08 (m, 8H, 4x OCH_2CH_3), 7.25 (d, *J* = 8.4, 4H, Ar*H*), 7.51 (d, *J* =8.0, 4H, Ar*H*), 8.31 (brs, N*H*). ^{13}C NMR (100 MHz, $CDCl_3$): δ (ppm) = 16.5 (d, *J* = 5.9, CH_3), 33.2 (d, *J* = 137.7, CH_2P), 62.4 (d, J_{CP} = 6.7, OCH_2), 121.3 (Ar:C), 127.7 (d, J_{CP} = 9.1, Ar:C), 130.3 (d, J_{CP} = 5.5, Ar:C), 136.3 (d, J_{CP} = 3.5, Ar:C), 163.6 (C Triazine), 167.8 (C-Cl Triazine). ^{31}P NMR (162 MHz, H_3PO_4/ $CDCl_3$): δ (ppm) = 26.4. *m/z* = 600 (MH^++2, 30%), 598 (MH^+, 100%). HRMS (ESI) *m/z* calcd for $C_{25}H_{35}ClN_5O_6P_2$ 598.17456 (^{35}Cl isotope) $[MH]^+$, found 598.17323.

Synthesis of tetraethyl ({[(6-chloro-1,3,5-triazine-2,4-diyl)bis(azanediyl)]bis(4,1-phenylene)}bis(hydroxymethylene))bis(phosphonate) (TP3)

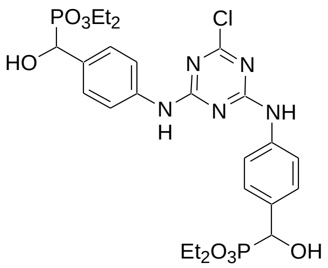

To a solution of diethyl (4-hydroxyphenyl)hydroxymethylphosphonate (**14**, 1.00 g, 3.857 mmol, 0.34 equiv) in THF (20 mL), under N_2, was added cyanuric chloride (**1**, 211 mg, 1.141 mmol) and DIPEA (2.04 mL, 11.641 mmol). The mixture was heated to reflux and stirred under N_2 overnight. The reaction mixture was cooled to room temperature and the solvent was removed in vacuum. The resulting oil was purified by column chromatography (1:1 ethyl acetate/acetone) to give compound **TP3** as a white solid (604 mg, 84%). mp 152-153 ^{o}C. ν_{max} (KBr) (cm^{-1}): 3376, 3269, 3200, 3110, 2988, 2931, 2908, 1618, 1572, 1524, 1508, 1434, 1419, 1396, 1314, 1302, 1233, 1213, 1181, 1124, 1096, 1044, 1022, 984, 863, 842, 801, 768, 731, 697, 637, 617, 561, 472. ^{1}H NMR (400 MHz, DMSO): δ (ppm) = 1.15 (t, *J* = 7.2, 6H,

2x OCH_2CH_3), 1.19 (t, *J* = 7.2, 6H, 2x OCH_2CH_3), 3.86-4.01 (m, 8H, 4x OCH_2CH_3), 4.90 (dd, *J* = 12.8 and *J* = 5.2, 2H, 2 x C*H*P(OH)), 6.18 (dd, *J* = 15.2 and *J* = 4.4, 2H, 2 x CHP(O*H*)), 7.38 (d, *J* = 7.2, 4H, Ar*H*), 7.62 (br s, 4H, Ar*H*). ^{13}C NMR (100 MHz, DMSO): δ (ppm) = 16.4 (dd, *J* = 7.4 and *J* = 5.2, *C*H_3), 61.5 (dd, J_{CP} = 34.6 and *J* = 7.0, O*C*H_2), 69.0 (d, *J* = 162.5, *C*HP(OH)), 120.2 (Ar:C), 127.6 (Ar:C), 133.6 (Ar:C), 137.6 (Ar:C), 163.8 (C Triazine), 168.2 (C-Cl Triazine). ^{31}P NMR (162 MHz, H_3PO_4/ DMSO): δ (ppm) = 21.8. *m/z* = 632 (MH^++2, 30%), 630 (MH^+, 100%). HRMS (ESI) *m/z* calcd for $C_{25}H_{35}ClN_5O_8P_2$ 630.16439 (Cl-35 isotope) $[MH]^+$, found 630.16318.

Synthesis of hexaethyl {[(1,3,5-triazine-2,4,6-triyl)tris(oxy)]tris(benzene-4,1-diyl)}tris(phosphonate) (TP4)

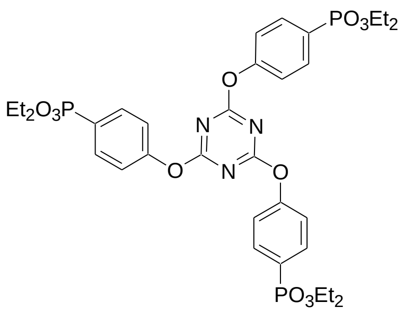

a) To a solution of diethyl (4-hydroxyphenyl)phosphonate (**2**, 309 mg, 1.342 mmol, 3.4 equiv) in THF (10 mL), under N_2, was added cyanuric chloride (**1**, 73 mg, 0.397 mmol) and DIPEA (0.7 mL, 4.050 mmol). The mixture was heated to reflux and stirred under N_2 overnight. The reaction mixture was cooled to room temperature and the solvent was removed in vacuum. The resulting oil was purified by column chromatography (1:1 ethyl acetate/acetone) to give compound **TP4** (53 mg, 17%) as white solid.

b) A mixture of diethyl 4-hydroxyphenylphosphonate (**2**, 256 mg, 1.112 mmol, 5.0 equiv), cyanuric chloride (**1**, 41 mg, 0.222 mmol) and Na_2CO_3 (685 mg) in toluene (30 mL) was heated to reflux and stirred under N_2 during 48 h. The reaction mixture was cooled to room temperature and the white solid was filtered and washed with hot EtOAc. After filtration, the solvent was removed under reduced pressure to afford an oil. The resulting oil was purified by column chromatography (1:1 EtOAc/acetone) to give compound **TP4** (129 mg, 76%) as white solid. mp 93-95 °C. ν_{max} (KBr) (cm^{-1}): 3096, 3066, 2988, 2942, 2911, 1602, 1570, 1496, 1445, 1370, 1232, 1207. ^{1}H NMR (400 MHz, $CDCl_3$): δ (ppm) = 1.34 (t, *J* = 7.2, 18H, 6x OCH_2CH_3), 4.05-4.14 (m, 12H, 6x OCH_2CH_3), 7.28 (dd, *J* = 8.6 and *J* = 3.4, 6H, Ar*H*), 7.85 (dd, *J* = 12.8 and *J* = 8.4, 6H, Ar*H*). ^{13}C NMR (100 MHz, $CDCl_3$): δ (ppm) = 16.4 (d, J_{CP} = 6.4, *C*H_3), 62.4 (d, J_{CP} = 5.5, O*C*H_2), 121.7 (d, J_{CP} = 15.9, Ar:C), 126.8 (d, J_{CP} = 190.4, Ar:C), 133.7 (d, J_{CP} = 11.9,

Ar:C), 154.4 (d, J_{CP} = 3.9, Ar:C), 173.3 (C Triazine). ^{31}P NMR (162 MHz, H_3PO_4/ $CDCl_3$): δ (ppm) = 17.4. MS (ESI): *m/z* = 766 (MH^+, 100%). HRMS (ESI) *m/z* calcd for $C_{33}H_{43}N_3O_{12}P_3$ 766.20541 $[MH]^+$, found 766.20331.

Synthesis of hexaethyl ({[(1,3,5-triazine-2,4,6-triyl)tris(oxy)]tris(benzene-4,1-diyl)}tris(methylene))tris(phosphonate) (TP5)

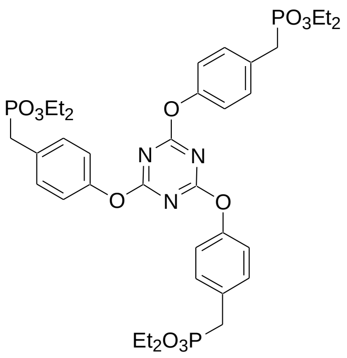

a) To a solution of compound **7** (500 mg, 2.047 mmol, 4 equiv) in THF (20 mL), under N_2, was added cyanuric chloride (**1**, 94 mg, 0.508 mmol) and DIPEA (0.5 mL, 2.56 mmol). The mixture was heated to reflux and stirred under N_2 overnight. The reaction mixture was cooled to room temperature and the solvent was removed in vacuum. The resulting oil was purified by column chromatography (1:1 ethyl acetate/acetone) to give compound **TP5** (104 mg, 25%) as white solid.

b) A mixture of compound **7** (345 mg, 1.413 mmol, 5.0 equiv), cyanuric chloride (**1**, 52 mg, 0.283 mmol) and Na_2CO_3 (870 mg) in toluene (40 mL), was heated to reflux and stirred under N_2 overnight. The reaction mixture was cooled to room temperature and the solid was filtered and washed with hot EtOAc. After filtration, the solvent was removed under reduced pressure to afford an oil. The resulting oil was purified by column chromatography (1:1 EtOAc:Acetone) to give compound **TP5** (164 mg, 72%) as white solid. mp 130-131 ^oC. ν_{max} (KBr) (cm^{-1}): 3077, 3051, 2985, 2932, 2912, 1608, 1581, 1508, 1479, 1444, 1383, 1367, 1243, 1217, 1178, 1090, 1055, 1024, 964, 861, 852, 805, 725, 631, 588, 534, 499, 430. ^{1}H NMR (400 MHz, $CDCl_3$): δ (ppm) = 1.23 (t, *J* = 7.2, 18H, 6x OCH_2CH_3), 3.14 (d, *J* = 21.6, 6H, 3x CH_2P), 3.94-4.07 (m, 12H, 6x OCH_2CH_3), 7.09 (d, *J* = 8.0, 6H, Ar*H*), 7.30 (dd, *J* =8.6 and *J* = 2.8, 6H, Ar*H*). ^{13}C NMR (100 MHz, $CDCl_3$): δ (ppm) = 16.5 (d, J_{CP} = 5.8, CH_3), 33.3 (d, J_{CP} = 138.3, CH_2P), 62.4 (d, J_{CP} = 7.1, OCH_2), 121.6 (d, J_{CP} = 2.6, Ar:C), 129.7 (d, J_{CP} = 9.5, Ar:C), 131.0 (d, J_{CP} = 6.1, Ar:C), 150.6 (d, J_{CP} = 4.2, Ar:C), 173.7 (C Triazine). ^{31}P NMR (162 MHz, H_3PO_4/ $CDCl_3$): δ (ppm) = 26.0. MS (ESI): *m/z* = 808 (MH^+, 100%). HRMS (ESI) *m/z* calcd for $C_{36}H_{49}N_3O_{12}P_3$ 808.25236 $[MH]^+$, found 808.24953.

Synthesis of dodecaethyl ({[(1,3,5-triazine-2,4,6-triyl)tris(oxy)]tris(benzene-4,1-diyl)}tris(methanetriyl))hexakis(phosphonate) (TP6)

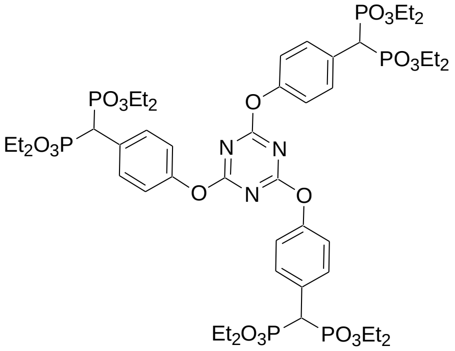

A mixture of compound **13** (330 mg, 0.868 mmol, 5.0 equiv), cyanuric chloride (**1**, 32 mg, 0.174 mmol) and Na_2CO_3 (670 mg) in toluene (75 mL), was heated to reflux and stirred under N_2 overnight. The reaction mixture was cooled to room temperature and the solid was filtered and washed with hot EtOAc. After filtration, the solvent was removed under reduced pressure to afford an oil. The resulting oil was purified by recrystallisation from DCM/n-hexane to give compound **TP6** (154 mg, 73%) as a white solid. mp 174-176ºC. ν_{max} (KBr) (cm^{-1}): 3066, 2988, 2937, 2910, 2874, 1609, 1577, 1507, 1477, 1458, 1444, 1420, 1380, 1246, 1216, 1182, 1165, 1064, 1022, 972, 892, 867, 810, 749, 639, 629, 608, 571, 546, 493. ^{1}H NMR (400 MHz, $CDCl_3$): δ (ppm) = 1.16 (t, *J* = 7.1, 18H, 6x OCH_2CH_3), 1.28 (t, *J* = 7.1, 18H, 6x OCH_2CH_3), 3.76 (t, *J* = 25.0, 3H, 3x P_2CH), 3.93-4.17 (m, 24H, 12x OCH_2CH_3), 7.14 (d, *J* = 8.4, 6H, Ar*H*), 7.52 (d, *J* =8.4, 6H, Ar*H*). ^{13}C NMR (100 MHz, $CDCl_3$): δ (ppm) = 16.3 (m, CH_3), 16.4 (m, CH_3), 45.2 (t, J_{CP} = 132.4, 3x P_2CH), 63.1 (m, OCH_2), 63.7 (m, OCH_2), 121.7 (Ar:C), 128.4 (t, J_{CP} = 7.2, Ar:C), 131.8 (t, J_{CP} = 6.3, Ar:C), 151.2 (t, J_{CP} = 3.1, Ar:C), 173.6 (C Triazine). ^{31}P NMR (162 MHz, H_3PO_4/ $CDCl_3$): δ (ppm) = 18.3. MS (ESI): *m/z* = 1216 (MH^+, 100%). HRMS (ESI) *m/z* calcd for $C_{48}H_{76}N_3O_{21}P_6$ 1216.33915 $[MH]^+$, found 1216.33949.

Attempt of synthesis of hexaethyl ({[(1,3,5-triazine-2,4,6-triyl)tris(oxy)]tris(benzene-4,1-diyl)}tris(hydroxymethylene))tris(phosphonate) (TP7) from 11

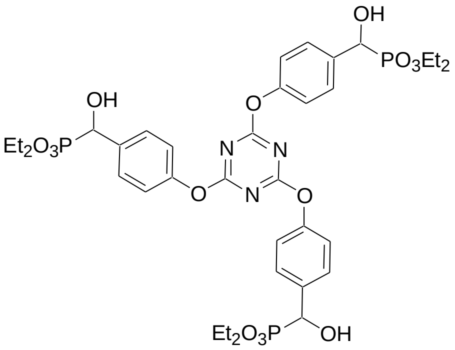

To a solution of diethyl [hydroxy(4-hydroxyphenyl)methyl]phosphonate (**11**, 206 mg, 0.792 mmol, 3.4 equiv) in THF (10 mL), under N_2, was added cyanuric chloride (**1**, 43 mg, 0.234 mmol) and DIPEA (0.21 mL, 1.17 mmol). The mixture was heated to reflux and stirred under N_2 overnight. The reaction mixture was cooled to room temperature and the solvent was removed in vacuum. The analysis of the ^{1}H NMR spectrum of the crude reaction mixture showed a 1:1.119 mixture of **TP7** and compound **11**, respectively, had been produced. Attempts of separation of these compounds by column chromatography did not afford a pure sample of the desired compound **TP7**.

NMR spectra of compound **TP7** (in the crude reaction mixture): ^{1}H NMR (400 MHz, DMSO): δ (ppm) = 1.15 (t, *J* = 7.2, 6H, 2x OCH$_2$C*H*$_3$), 1.19 (t, *J* = 6.8, 6H, 2x OCH$_2$C*H*$_3$), 3.95 -4.03 (m, 8H, 4x OC*H*$_2$CH$_3$), 5.01 (d, *J* = 13.6, 2H, 2 x C*H*P(OH)), 7.26 (d, *J* = 8.4, 4H, Ar*H*), 7.52 (dd, *J* = 8.8 and *J* = 2.0, 4H, Ar*H*). ^{13}C NMR (100 MHz, DMSO): δ (ppm) = 16.3 (m, *C*H$_3$), 61.9 (O*C*H$_2$), 62.3 (O*C*H$_2$), 68.8 (d, *J* = 162.1, *C*HP(OH)), 120.7 (Ar:C), 128.7 (Ar:C), 136.7 (Ar:C), 150.5 (Ar:C), 172.0 (C Triazine). ^{31}P NMR (162 MHz, H_3PO_4/ DMSO): δ (ppm) = 21.5. MS (ESI): *m/z* = 856 (MH$^+$, 100%). HRMS (ESI) *m/z* calcd for $C_{36}H_{49}N_3O_{15}P_3$ 856.23710 [MH]$^+$, found 856.23569.

Synthesis of 4,4',4''-[(1,3,5-triazine-2,4,6-triyl)tris(oxy)]tribenzaldehyde (19)

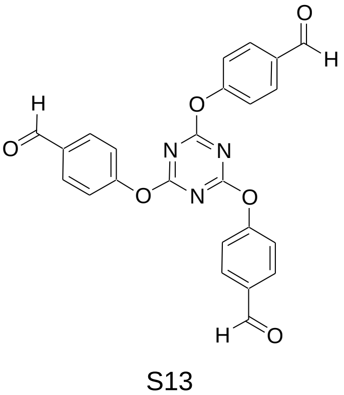

A mixture of 4-hydroxybenzaldehyde (**12**, 2.40 g, 19.65 mmol), cyanuric chloride (**1**, 900 mg, 4.88 mmol) and Na_2CO_3 (15.0 g) in toluene (75 mL), was heated to reflux and stirred overnight under N_2. The reaction mixture was cooled to room temperature and the white solid was filtered and washed with hot EtOAc. The filtrated solution was washed with 10% aqueous Na_2CO_3 solution twice. The combined organic extracts were washed with H_2O and dried over anhydrous $MgSO_4$. After filtration, the solvent was removed under reduced pressure to afford compound **19** [9] (1.87 g, 87%) as a white solid. ^{1}H NMR (400 MHz, $CDCl_3$): δ (ppm) = 7.33 (d, *J* = 8.2, 6H, Ar*H*), 7.93 (d, *J* = 8.4, 6H, Ar*H*), 10.00 (s, 3H, 3x ArC*H*O).

Attempt of synthesis of hexaethyl ({[(1,3,5-triazine-2,4,6-triyl)tris(oxy)]tris(benzene-4,1-diyl)}tris(hydroxymethylene))tris(phosphonate) (TP7) from 19

A mixture of compound **19** (200 mg, 0.453 mmol), diethyl phosphonate (0.2 mL, 1.495 mmol) and triethylamine (0.21 mL, 1.495 mmol) in toluene (10 mL) was stirred at room temperature overnight. The reaction mixture was acidified with 10% aqueous HCl solution and the aqueous layer was extracted with EtOAc. The combined organic extracts were washed with brine and dried over anhydrous $MgSO_4$. After filtration, the solvent was removed under reduced pressure. The resulting oil was purified by column chromatography (1:1 EtOAc/acetone) to give compound **20** (35 mg, 13%) as white solid. mp 88-90 °C. ν_{max} (KBr) (cm^{-1}): 3386, 3072, 2985, 2930, 2909, 2850, 2834, 2742, 1702, 1604, 1593, 1572, 1503, 1475, 1466, 1459, 1420, 1375, 1300, 1216, 1163, 1019, 970, 843, 808, 620, 561, 503. ^{1}H NMR (400 MHz, $CDCl_3$): δ (ppm) = 1.21-128 (m, 6H, 2x OCH_2CH_3), 4.00 (m, 4H, 2x OCH_2CH_3), 5.02 (d, *J* = 11.2, 1H, C*H*P(OH)), 7.12 (d, *J* = 8.0, 2H, Ar*H*), 7.32 (d, *J* = 8.0, 4H, Ar*H*), 7.50 (d, *J* = 8.0, 2H, Ar*H*), 7.91 (d, *J* = 8.0, 4H, Ar*H*), 9.94 (s, 2H, 2x ArC*H*O). ^{13}C NMR (100 MHz, $CDCl_3$): δ (ppm) = 16.5 (m, CH_3), 63.5 (m, OCH_2), 70.3 (d, *J* = 158,7, *C*HP(OH)), 121.3 (d, J_{CP} = 2.1, Ar:C), 122.4 (Ar:C), 128.5 (d, J_{CP} = 5.6, Ar:C), 131.4 (Ar:C), 134.4 (Ar:C), 135.0 (d, J_{CP} = 2.0, Ar:C), 151.2 (m, Ar:C), 155.9 (Ar:C), 173.3 (C Triazine), 173.9 (C Triazine),191.0 (Ar:*C*HO). ^{31}P NMR (162 MHz, H_3PO_4/ $CDCl_3$): δ (ppm) = 20.8. MS (ESI): *m/z* = 580 (MH^+, 100%). HRMS (ESI) *m/z* calcd for $C_{28}H_{27}N_3O_9P$ 580.14794 $[MH]^+$, found 580.14692.

1.5. Membrane Preparation

Membranes were prepared by a casting method using Nafion®/DMAc solutions, based on our previous works, using 1 wt% loading of **TP** dopants. The 20 wt% Nafion solution was dried under reduced pressure, at 40 °C, until a dry residue was obtained. A new 10 wt % solution of Nafion was obtained by dissolution of the dried Nafion in the required amount of *N,N*-dimethylacetamide (DMAc).The TP dopant quantity was added to DMAC solution and the mixture was stirred during 1–5 h, in an ultrasonic bath, to guarantee the complete dissolution of dopants. The resulting solutions were casted on a Petri dish and slowly evaporated, until obtaining homogeneous membranes. The resulting membranes were dried in a vacuum oven at 60 °C, and were followed by their annellation for 2 h, at 140 °C. The membranes were activated by a sequential treatment, with 1 h for each step, by boiling them in H_2O_2 solution (3%), washing with hot deionized water, boiling in a 0.5 M sulfonic acid solution, and washing again with hot deionized water. After activation, the membranes were kept in deionized water until their use. The new membranes were labelled as Nafion/TP*i*-1.0, respectively, where ***i*** indicates the specific triazine used, and 1.0 specifies the wt% of dopant. Recast Nafion films were also prepared, for comparison, without the incorporation of TPs.

Doped and recast Nafion membranes had an average thickness of 94 ± 6 µm.

1.6. FTIR-ATR spectra of the new membranes

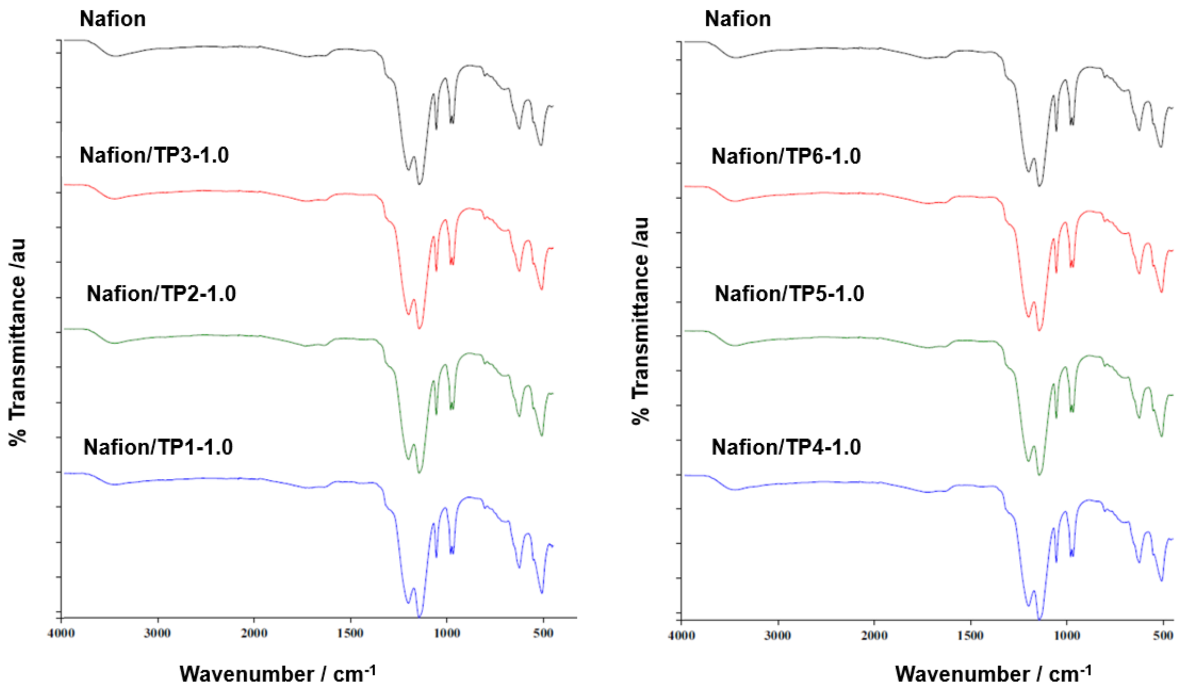

Figure S1: FTIR-ATR spectra of the new membranes.

2. References

1. Goossen, L. J.; Dezfuli, M. K. *Synlett* **2005**, 445-448. doi:10.1055/s-2005-862372
2. Kim, Y. C.; Brown, S. G.; Harden, T. K.; Boyer, J. L.; Dubyak, G.; King, B. F.; Burnstock, G.; Jacobson, K. A. *J. Med. Chem.* **2001**, *44*, 340-349. doi:10.1021/jm9904203
3. Chow, H. F.; Mak, C. C. *J. Org. Chem.* **1997**, *62*, 5116-5127. doi:10.1021/jo970383s
4. Boyer, S. H.; Jiang, H. J.; Jacintho, J. D.; Reddy, M. V.; Li, H. Q.; Li, W. Y.; Godwin, J. L.; Schulz, W. G.; Cable, E. E.; Hou, J. Z.; Wu, R. R.; Fujitaki, J. M.; Hecker, S. J.; Erion, M. D. *J. Med. Chem.* **2008**, *51*, 7075-7093. doi:10.1021/jm800824d
5. Rao, K. U. M.; Sundar, C. S.; Prasad, S. S.; Rani, C. R.; Reddy, C. S. *Bull. Korean Chem. Soc.* **2011**, *32*, 3343-3347. doi:10.5012/bkcs.2011.32.9.3343
6. Vovk, A. I.; Kalchenko, V. I.; Cherenok, S. A.; Kukhar, V. P.; Muzychka, O. V.; Lozynsky, M. O. *Org. Biomol. Chem.* **2004**, *2*, 3162-3166. doi:10.1039/b409526j
7. Teixeira, F. C.; de Sa, A. I.; Teixeira, A. P. S.; Rangel, C. M. *Appl. Surf. Sci.* **2019**, *487*, 889-897. doi:10.1016/j.apsusc.2019.05.078

8. Németh, G.; Greff, Z.; Sipos, A.; Varga, Z.; Székely, R.; Sebestyén, M.; Jászay, Z.; Béni, S.; Nemes, Z.; Pirat, J. L.; Volle, J. N.; Virieux, D.; Gyuris, A.; Kelemenics, K.; Ay, E.; Minarovits, J.; Szathmary, S.; Kéri, G.; Orfi, L. *J. Med. Chem.* **2014**, *57*, 3939-3965. doi:10.1021/jm401742r

9 Tahmassebi, D. C.; Sasaki, T. *J. Org. Chem.* **1994**, *59*, 679-681. doi:10.1021/jo00082a034
